# Role of Environmental Quality of Life in Physical Activity Status of Individuals with and without Physical Disabilities in Saudi Arabia

**DOI:** 10.3390/ijerph19074228

**Published:** 2022-04-01

**Authors:** Aqeela Zahra, Muhammad Shehzad Hassan, Jae-Hyun Park, Sehar-un-Nisa Hassan, Nuzhat Parveen

**Affiliations:** 1 Department of Family and Community Medicine, College of Medicine, University of Ha’il, Ha’il 81451, Saudi Arabia; 2 College of Medical Rehabilitation Sciences, Taibah University, Madinah 42353, Saudi Arabia; mshhassan@taibahu.edu.sa; 3 Department of Social and Preventive Medicine, School of Medicine, Samsung Biomedical Research Institute, Sungkyunkwan University, 2066 Seobu-ro, Jangan-gu, Suwon 440-746, Korea; pjaehyun@skku.edu; 4 College of Public Health and Health Informatics, University of Ha’il, Ha’il 81451, Saudi Arabia; s.nisa@uoh.edu.sa; 5 Department of Obstetrics and Gynecology, College of Medicine, University of Ha’il, Ha’il 81451, Saudi Arabia; n.parveen@uoh.edu.sa

**Keywords:** disabled, physical activity, environmental quality of life, predictors of physical activity, Saudi Arabia

## Abstract

The promotion of physical activity (PA) in various subgroups of the population such as people with physical disabilities has been spotlighted in the revised guidelines of The World Health Organization (WHO), Geneva, Switzerland. In order to update public health interventions, there is a need to identify factors that may promote or prevent engagement in PA for special subgroups of the population. This study aims to calculate the PA levels of individuals with and without physical disabilities in Saudi Arabia, their assessment of the environmental (EQoL), and the predictive role of EQoL in PA. The International Physical Activity Questionnaire (IPAQ) and the EQoL domain in standardized WHOQoL Questionnaire were administered on both groups of the population. The study sample comprised 116 individuals with physical disabilities and 243 individuals without any form of disability as a control group. A regression analysis was performed to analyze the predictors of PA in both groups. Findings showed that among the individuals with disabilities, older people were more likely to engage in PA as compared to the younger age group (*p* < 0.05) and males were significantly less likely to meet the PA criteria. Some of the EQoL features such as safety increased the likelihood of PA up to 2.3 times (*p* < 0.05) in individuals with physical disabilities. In addition, opportunities for leisure activities were a significant predictor of PA among both groups of individuals with and without physical disabilities (*p* < 0.05). Our findings suggest that upcoming public health interventions should focus on improving various dimensions of EQoL for the promotion of physical activity among individuals with physical disabilities. Additional studies are needed to further explore various sociodemographic and environmental factors which can affect the PA status of disabled groups.

## 1. Introduction

According to a global report on disabilities, 15% of the world’s population (over a billion people) is living with some kind of disability [1]. Physical disability is one of the prevailing types of disability in children and adults. The Americans with Disabilities Act defines physical disability as an impairment that markedly limits the activities of daily life [2]. In Saudi Arabia, disability is a substantial social and economic issue [3]. According to national demographic survey data, more than half a million people (1 in every 30 persons) in Saudi Arabia were living with some kind of disability in 2016, and this number increased to 1.4 million in 2021 [4]. The latest statistics have revealed a disability-prevalence rate of 7.1%, with physical disability affecting the highest number of individuals (3.9%) in Saudi Arabia [5]. However, these rates are expected to increase due to a continuous rise in health risk factors such as obesity, physical inactivity, road traffic accidents, and chronic diseases. The increasing number of people with disabilities is a constant challenge for the Saudi government and health care authorities. The provision of suitable living environment, access to education, adoption of healthy lifestyle, and leisure activities can improve the quality of life (QOL) of disabled individuals and substantially decrease the burden on health system [6].

Many previous studies prove that regular physical activity (PA) has many beneficial effects for both disabled and non-disabled individuals [7,8]. Some researchers suggest that PA is even more beneficial for disabled people in improving their physical, psychological, and emotional well-being [9]. Most physically disabled individuals do not perform even the minimum level of PA recommended by World Health Organization (WHO) [10]. This inactivity can further increase their risk of suffering from chronic diseases and worsen their overall health.

There are a number of barriers that can reduce the level of PA among people. The surrounding environment and neighborhood aesthetics play an important role in encouraging individuals to participate in PA [11]. Easy access to public parks and gyms with special facilities for disabled people can also increase their PA status [12]. Therefore, it is important to explore the environmental quality of general as well as the disabled population and its relationship with PA performance.

Previous research studies in Saudi Arabia mostly focused on calculating the prevalence, severity, and types of disability [13,14]. These studies reported a disability-prevalence rate of 376 per 1000 among children, with traumatic accidents being the most important cause of physical disability among young males. Some studies analyzed the status of other challenges such as transport and education for disabled [15,16]. Saeed Almaki reported that transportation services are a major hindrance for the movement of special needs people, and education and transport services must be improved to raise their living standard [15,16]. General quality of life was also assessed for disabled individuals in some scientific papers [17,18]. A few articles calculated the prevalence and predictors of PA in Saudi Arabia among non-disabled people [19,20,21]. According to these studies, only 17.4% of Saudis performed PA, and their PA status was reduced drastically due to COVID-19 pandemic. However, to our knowledge, there is no previous study in Saudi Arabia that assessed the prevalence of PA among disabled people and identified the factors affecting it.

Therefore, in this study, we aim to calculate and compare the PA of disabled and non-disabled individuals in the context of fulfilling the WHO’s recommendations. We will also asses the environmental EQoL of both groups. Finally, this study will also investigate the relationship of age, gender, and EQoL with the PA status of disabled and non-disabled individuals.

## 2. Materials and Methods

### 2.1. Study Population

Our study population included individuals with and without disabilities. All of the people included in this study were Saudi citizens currently living in Saudi Arabia. The study sample consisted of both males and females who were 15 years of age or above. Among the disabled, only people with physical disabilities were included in this study. The total sample in this study consisted of 359 participants. Non-disabled people accounted for 67.7%, while disabled people composed 32.3% of the total sample. Of the participants, 23.3% had a mild disability, 61.2% had a moderate disability, and 15.5% had a severe disability.

### 2.2. Procedure

Data for the current study was collected from October to December 2021. Information for disabled individuals was collected from the Rehabilitation Hospital Madinah. Madinah is one of the major metropolitan cities in Saudi Arabia. This hospital provides rehabilitation services to people with any kind of physical disability, including stroke and amputation. It has an inpatient department for patients with long term disabilities. Physiotherapy, prosthetics, and orthotics departments are also available in this hospital. Individual face-to-face interviews were conducted to collect the data from disabled individuals using structured survey questionnaire. As all of the study participants were Arabic speakers, interviews were conducted in Arabic. Detailed informed consent was taken, and the purpose of the study was explained to the participants before the interview. The interviews were conducted by hospital internees in the department of physical rehabilitation who were trained to collect data on study tools. Physical disability is defined as any condition of the body that makes it more difficult for the person with the condition to do certain activities and interact with the world around them. Participants with physical disability were categorized according to the severity of disability as mild, moderate, and severe. Data about the severity level were collected from electronic medical records where the patients were already classified by their attending physician.

Keeping in view the social distancing protocols implemented during the COVID-19 pandemic, data from non-disabled people was collected through an online questionnaire on Google, which was distributed through social media platforms. The World Health Organization Quality of Life (WHOQOL-BREF) questionnaire and International Physical Activity Questionnaire Short Form (IPAQ-SF) were used for data collection.

### 2.3. Measures

#### 2.3.1. World Health Organization Quality of Life (WHOQOL-BREF) Questionnaire

In order to assess the quality of life (QOL) among the sample population (both disabled and non-disabled), the World Health Organization Quality of Life (WHOQOL-BREF) questionnaire was used. This questionnaire contains 24 items to assess the QOL in four different domains including physical health, psychological health, social relationships, and the environment. In our study, only the environmental QOL domain was used, which includes eight items questionnaire. The Arabic version of the questionnaire was included for data collection [22]. The Arabic version of the WHOQOL-BREF has well-established reliability and validity [23,24], and it has been extensively used previously to assess QOL among people with physical disabilities as well as in the general population [25,26,27].

EQoL was assessed using the eight-item questionnaire included in WHOQOL-BREF. These eight items inquired about financial resources, freedom, physical safety, healthcare accessibility and quality, home environment, opportunities for acquiring new information, opportunities for leisure activities, the physical environment (pollution/noise/traffic/climate), and transport. The satisfaction level with each item was assessed on a 5-point Likert scale. Higher points indicated better item score.

#### 2.3.2. International Physical Activity Questionnaire Short Form (IPAQ-SF)

The level of PA among the participants was assessed using the International Physical Activity Questionnaire Short Form (IPAQ-SF). This questionnaire is a reliable tool with acceptable validity and reliability [24,28] to measure the type, frequency (measured in days per week), and duration (time per day) of PA by an individual. It contains seven questions about the vigorous, moderate and mild physical activity and sitting time in the last 7 days. Participants were asked, “during the last 7 days, on how many days did you do physical activity”? They were also asked about the duration of PA in minutes and hours. Both these questions were asked separately for mild, moderate, and vigorous activity.

Metabolic equivalent of task (MET)-minutes were used to assess the amount of PA per week. MET is a multiple of resting energy expenditure. One MET is the amount of energy spent while at rest. Thus, individuals with more PA have a higher number of MET-minutes. The MET-minutes/week were calculated for each participant by adding the individual MET-minutes in each type of exercise (mild, moderate, severe). Detailed methodology to calculate MET-minutes is available on the IPAQ website [29]. According to the WHO recommendations, an individual should have at least 600 MET-minutes per week to maintain good health [6]. We categorized our study population into two groups; those meeting the WHO recommendations for PA (more than 600 MET-minutes/week) and the ones not meeting WHO recommendations for physical activity (less than 600 MET-minutes/week).

### 2.4. Statistical Analysis

Statistical analysis was executed using the IBM SPSS software version 21.0. Univariate analysis was performed to analyze the distribution of the sample according to demographic and health-related variables. Bivariate analysis (Chi-square test) was performed to assess the PA status of individuals according to background variables and disability status. The EQoL of disabled and non-disabled individuals was analyzed by an independent sample *t*-test. The relationship between PA status and EQoL among disabled and non-disabled individuals was examined by binary logistic regression analysis (multivariate analysis). The strength of the relationship between these variables was expressed as odds ratios (ORs). A *p* value of less than 0.05 was taken to be statistically significant.

### 2.5. Ethical Considerations

The study was conducted in accordance with the Declaration of Helsinki and approved by the Institutional Review Board of the University of Ha’il, Ha’il, Saudi Arabia, dated 13 December 2021 and approved by the university letter H-2021-229. The informed consent form was also reviewed in detail by the ethical approval committee. Informed consent was obtained from all subjects involved in the study after the aim of study and the privacy of personal information were explained.

## 3. Results

### 3.1. Background Characteristics

The total sample in this study consisted of 359 participants. Supplementary Table S1 shows the background characteristics of the study population. Sixty-eight percent of the participants in this study were females, and most of the individuals were between 15 and 35 years of age (59.6%). Among the participants, 50.7% were unmarried and 44.6% of the total sample had completed college or university education. Non-disabled people accounted for 67.7%, while disabled people composed 32.3% of the total sample.

### 3.2. PA Status of the Individuals According to Background Variables

The overall status of PA according to background variables is displayed in Supplementary Table S1. According to our analysis, 54% of the individuals did not meet the WHO criteria for minimum PA per week. People who completed the criteria for PA dominantly included females (64.8%), young individuals (56.4%), and people who had a primary or secondary education level (50%). A higher number of participants with no disability (65.5%) or chronic disease (71.5%) met the recommendation for PA.

Table 1 shows the PA of disabled and non-disabled people according to age and gender. The bivariate analysis demonstrates that among individuals with disabilities, a significantly higher proportion of females met the MET criterion for PA (*p* < 0.01), whereas among individuals without disabilities, a significantly higher proportion of male participants fulfilled the MET criterion for PA (*p* < 0.001). The comparison across age groups shows that a higher proportion of elder individuals with disabilities did not meet the MET criterion (*p* < 0.05), and a significantly higher proportion of individuals without disabilities achieved the MET criterion for PA (*p* < 0.5). Of the disabled people, 40.4% who had a chronic disease met the PA criteria, while of people who were not disabled but had a chronic disease, 70.3% were physically active (*p* < 0.01).

### 3.3. Comparison of EQoL of Individuals with and without Disabilities

Mean scores of EQoL in the total sample and according to disability status are shown in Table 2. Overall EQoL was highest with daily life safety followed by satisfaction with transport and access to health care services. Mean scores of satisfaction with safety in daily life (*p* < 0.01), availability of day-to-day information (*p* = 0.03), and transport services were significantly higher in the non-disabled as compared to the disabled group. Disabled individuals reported a higher level of satisfaction with a healthy physical environment (*p* < 0.01), financial resources, opportunities for leisure activities (*p* = 0.01), and access to healthcare services as compared to non-disabled people.

### 3.4. Association of Environmental QOL, Age, and Gender with Physical Activity Status of Individuals with and without Disabilities

The relationship between age, gender, and environmental QOL with PA status is displayed in Table 3. Among the disabled population, older people met the WHO criteria for PA significantly less (OR = 0.39) than the younger group (*p* = 0.04). In contrast to the disabled group, the older population in the non-disabled group met the standard PA level 4.7 times more compared to young adults (*p* = 0.02). Males were significantly less likely to meet the PA criteria than females in the disabled sample (OR = 0.33) as compared to the non-disabled sample, where males had three times higher odds of fulfilling the PA recommendations (*p* < 0.01).

In the disabled sample, individuals who reported higher satisfaction level with environmental safety had 2.3 times more odds of meeting PA criteria as compared to the ones who reported less contentment with daily life safety (*p* = 0.04). Similarly, a higher satisfaction level with opportunities for leisure activities was also a significant predictor (*p* < 0.01) of standard PA level among the disabled group (OR = 2.2).

In the non-disabled population, participants who reported higher satisfaction with availability of daily life information (OR = 1.56) and opportunities of leisure activities (OR = 1.36) had significantly higher chances of achieving the PA criteria of WHO for optimum health as compared to the ones who reported a lower satisfaction level.

## 4. Discussion

Accelerated rates of chronic diseases in developing countries such as Saudi Arabia emphasize a change in health behavior. PA has proven effects on both the prevention and the control of many health related risk factors [30]. Along with healthy individuals, special population groups with disabilities also need to be encouraged to incorporate PA into their daily routine, which can help to improve their physical and psychological QOL [9,31]. A number of environmental factors, including access to transport, provision of parks and sports facilities, and a physical atmosphere play a significant role in encouraging people to adopt healthy life style and PA [32,33]. This study analyzed the PA level of disabled and non-disabled population in Saudi Arabia and its relationship with EQoL.

Our results show that more than half of the individuals (54%) in the study sample did not meet the WHO recommendations for PA. In fact, 14.5% of the total individuals were not doing any type of PA at all. These findings are consistent with the previous studies, which also show high levels of physical inactivity in Saudi Arabia [21,34]. This prevalence of physical inactivity is higher than many other countries in Europe and Asia [35,36,37]. A study in China reported 66.3% prevalence of PA, which is much higher than in Saudi Arabia [36]. Similarly, a recent study in Nepal reported that 95% of the population was physically active [37]. These findings suggest further exploration of factors affecting the PA level and the formulation of policies that can facilitate and encourage an active environment.

In our analysis, the prevalence of optimum PA was significantly higher in females (65.9%, *p* < 0.01) and younger age groups (56.6%, *p* = 0.04) among disabled people, while males (71.4%, *p* < 0.01) and older age group were more active in the non-disabled group (76.5%, *p* = 0.02). These findings were supported by the regression analysis as well. Previous studies in Saudi Arabia also suggest a high physical inactivity level among females, which contributes to an increased health burden and morbidity rates [21]. A lower number of fitness clubs and gyms for females are one of the contributing factors in decreased PA [38]. This difference in prevalence rates among males and females must be considered while planning PA promotion campaigns and the built environment. More facilities should also be provided at fitness centers to men with disabilities to promote PA among them. Younger adults with disabilities may be more physically active due to their regular contact with healthcare providers and awareness about their ill health. On the other hand, youth without any disability or chronic condition may have less knowledge about the importance of PA even in healthy individuals. These findings advocate the design and implementation of health education programs for specific age groups among disabled and non-disabled populations. Our analysis also shows that people with disability and co-existing chronic disease had significantly lower prevalence of PA (40.4%) as compared to the non-disabled with chronic disease (70.3%). This can be explained by the fact that chronic diseases superimposed on disability make it even more difficult to perform PA. Provision of physical therapy and exercise facilities at hospitals and rehabilitation centers for disabled can help in improving the health outcomes.

Assessment of the EQoL revealed that our sample population had the lowest mean score for satisfaction with facilities for leisure activities overall as well as separately among disabled and non-disabled groups. In terms of the built environment, public parks, sidewalks, and sport facilities play an important role in developing a physically active society [39]. In the previous few years, the Saudi Ministry of Health and Ministry of Education has launched a number of initiatives such as an increase in the number of parks, the development of safe and suitable walking tracks, and the implementation of health education of the public by physicians. Increasing the participation of community in sports and PA is also an important objective of the kingdom of Saudi Arabia’s Vision 2030 [38]. In spite of all these efforts, common people seem to be less satisfied with PA facilities. A previous study’s analysis showed that the initiatives by Saudi ministries were mostly short-term and fragmented, and the information about such programs was not properly disseminated, which may have contributed to dissatisfaction of people with the leisure opportunities [38]. The establishment of a central authority that can design more sustained and integrated health promotion campaigns and provision of gyms and public parks near residential areas with specific facilities for special population groups can help to promote PA.

Our analysis also revealed that disabled people were significantly less satisfied with the safety of daily life (*p* < 0.01) and the availability of day-to-day information (*p* = 0.03) as compared to the non-disabled group. These results can contribute in policy implications for people with disabilities. The provision of new and updated information, especially in the context of the COVID-19 pandemic, can substantially promote the QOL of individuals with specific needs. Specific arrangements such as elevators and rails for disabled people at public places can contribute in their increased satisfaction with the safety of daily life.

Regression analysis in our study showed that among disabled individuals, those who were more satisfied with the safety of daily life (OR = 2.32), access to daily information (OR = 2.12), and opportunities for leisure activities (OR = 2.21) were significantly more likely to meet the WHO recommendations for PA. The non-disabled group also had a significant positive association of PA with the provision of day-to-day information (OR = 1.56) and facilities for leisure activities (OR = 1.36), but there was no association with the safety of daily life. Many previous studies suggest that safety from crimes, transport safety, and overall easy and safe access to recreation facilities and public parks has a positive association with PA level among communities [40,41]. In general, the crime rate in Saudi Arabia is low, which may have contributed to the non-association of environmental safety with PA levels among the non-disabled population [42]. However, other aspects of safety such as transport facilities, wheelchair access services, and specific walking tracks are of more concern for the disabled population, resulting in stronger significant association with their PA status. Our findings also suggest that provision of health-related information, social media campaigns, and increasing public health education can play a stronger role in increasing the PA level specifically among the disabled. Upgrading PA opportunities and designing them to meet the needs of special population groups can also further encourage PA among the Saudi population.

### Limitations and Direction for Future Research

This study has a few limitations. Firstly, our total sample size is relatively small, but it is still comparable to previous studies. Collecting data during the COVID-19 pandemic was an additional challenge, which may have hindered the generalizability of the results. Secondly, we included people with physical disabilities only, which may have limited the variation in our results. People with intellectual, visual, or other types of disabilities may have different PA levels and environmental QOL. Thirdly, the PA levels were self-reported by the individuals. It is highly probable that people may wrongly perceive their PA as vigorous or highly intensive. Fourthly, the proportion of males and females was not equal in the study sample, which may have affected the results. Our study also lacks a proper sampling frame and random selection due to the limitation of resources, which may cause bias in the results. Future studies with large datasets, proper sampling frame, and stratification and inclusion of people with different types of disabilities could help in providing broader knowledge about the current situation of PA among special needs groups. A more objective evaluation of PA level by asking additional questions such as heart rate or respiratory rate may provide the exact estimates of activity.

## 5. Conclusions

Our research findings underscore the need to improve the EQoL to promote PA in various subgroups of the population such as people with physical disabilities. Among the various aspects of EQoL, safety features were found to be significant factors for individuals with disabilities. Ensuring the daily life safety of disabled people by providing wheelchair services, elevators, side rails, and special toilets can also contribute to improving their PA level.

The other significant predictor of PA in individuals with disabilities and without disabilities was reduced access to leisure activities. The data for this study were primarily collected from Medina city, which is a prime place of attraction for pilgrims who visit Prophet Mosque and several other places in the city due to their historical and religious importance. It is quite probable that for the local population, there are limited opportunities for leisure activities in the form of public parks and sidewalks. The local authorities can review the situation and strategies such as city expansion and improving the EQoL through provision of walkways, parks, gyms, and fitness clubs.

The overall low engagement in PA is also attributable to social norms and cultural factors in Saudi Arabia, where sedentary life is preferred and most entertainment and outdoor activities revolve around socialization and eating [43]. There is an overall culture of comfort, PA is not preferred, and public spaces for instance streets are not considered suitable for PA. Additionally, the extremely hot climate, built environment, and inadequate public transportation systems also discourage outdoor PA [43,44]. A systematic review shows that a lower level of PA among women is due to specific gender norms, including specific dressing for women in public places, decreased accessibility to gyms as women are less likely to drive cars, and less social and family support [44,45].

All these cultural and social factors must be taken into account while designing and implementing PA recommendations. Collaborative efforts including ministries of health, sports, and urban planning should be made to encourage PA, separate gym facilities for females should be provided, and health education should be encouraged among families to give equal importance to PA for females. Furthermore, the overall culture of sedentary lifestyle and unhealthy eating should be discouraged by health awareness campaigns.

In addition, examples of strategies and actions can be adopted to devise inclusive PA programs. For example, China launched a National Fitness Program, which included the promotion of PA through providing safe and clean environment; constructing accessible and walkable places; and facilitating workplaces, schools, and rural areas for PA [46]. Similarly, the Nepal government launched yoga programs at schools and workplaces and introduced sports and PA through public events [37]. These interventions, if adapted in accordance with social norms and local context through more representation of community in design and implementation, could have more effective results.

It is important to develop a strategic framework to enhance the environmental QoL for all sections of the population with special focus on people with disabilities. Future studies exploring gender, age, and cultural factors can provide a better understanding and further insight for policy designing and interventions to promote PA among special population groups.

## Figures and Tables

**Table 1:** Physical activity status of the disabled and non-disabled individuals across gender and age (Chi-square, Bivariate analysis).

Background Variables	Disabled *n* = 116			Non-Disabled *n* = 243		
	MET-Minutes Less than 600 ^1^	MET-Minutes More than 600 ^2^	*p* Value	MET-Minutes Less than 600 ^1^	MET-Minutes More than 600 ^2^	*p* Value
	*n* (%)	*n* (%)		*n* (%)	*n* (%)	
Gender			<0.01 *			<0.001 *
Males	44 (61.1)	28 (38.9)		12 (28.6)	30 (71.4)	
Females	15 (34.1)	29 (65.9)		123 (61.2)	78 (38.8)	
Age (years)			0.05 *			<0.01 *
Young (15–35)	23 (43.4)	30 (56.6)		98 (60.9)	63 (39.1)	
Middle age (36–55)	13 (44.8)	16 (55.2)		33 (50.8)	32 (49.2)	
Elder (55+)	23 (67.6)	11 (32.4)		4 (23.5)	13 (76.5)	

^1^ Did not meet the WHO recommendation for physical activity; ^2^ Met the WHO recommendation for physical activity; * significant *p*-value.

**Table 2:** Mean scores on EQoL in total sample and between individuals with and without disabilities (*t*-test).

Items Assessing Environmental QOL	Mean Score ^1^ of EQoL		
	Total Mean (S.D)	Non-Disabled Mean (S.D)	Disabled Mean (S.D)
How safe do you feel in your daily life?	4.17 (0.89)	4.31 ** (0.92)	3.87 (0.75)
How healthy is your physical environment?	3.71 (1.01)	3.57 (1.08)	4.01 ** (0.75)
Have you enough money to meet your needs?	3.73 (1.06)	3.69 (1.18)	3.81 (0.75) _(ns)_
How available to you is the information that you need in your day-to-day life?	3.82 (0.92)	3.89 * (0.98)	3.67 (0.75)
To what extent do you have the opportunity for leisure activities?	3.27 (1.15)	3.17 (1.16)	3.47 ** (1.10)
How satisfied are you with the conditions of your living place?	3.97 (0.99)	3.96 (1.11)	3.97 (0.70) _(ns)_
How satisfied are you with your access to health services?	4.03 (1.04)	4.02 (1.14)	4.04 (0.80) _(ns)_
How satisfied are you with your transport services?	4.10 (0.93)	4.14 (1.00)	4.02 (0.74) _(ns)_

^1^ Total score for each item is 5; * significant *p*-value; *p*-value significance ** *p* < 0.01, * *p* < 0.05; _(ns)_ Not Significant.

**Table 3:** Predictive role of EQoL in physical activity status of individuals with disabilities and without disabilities (Binary logistic regression, Multivariate Analysis).

Predictor Variables	Categories	OR (95% CI) ^1^ Disabled	OR (95% CI) ^1^ Non-Disabled
Age	Young (Ref)		
	Middle age	1.10 (0.42–2.84)	1.61 (0.84–3.08)
	Elder	0.39 * (0.15–1.00)	4.74 * (1.25–17.88)
Gender	Females (Ref)		
	Males	0.33 ** (0.14–0.74)	3.03 ** (1.37–6.70)
Environmental Quality of Life (EQoL)	How safe do you feel in your daily life?	2.32 * (1.02–5.25)	1.19 (0.80–1.77)
	How healthy is your physical environment?	1.41 (0.62–3.22)	0.96 (0.68–1.35)
	Have you enough money to meet your needs?	0.96 (0.42–2.20)	0.84 (0.61–1.14)
	How available to you is the information that you need in your day-to-day life?	2.12 (0.93–4.86)	1.56 ** (1.07–2.27)
	To what extent do you have the opportunity for leisure activities?	2.21 * (1.24–3.96)	1.36 * (1.02–1.80)
	How satisfied are you with the conditions of your living place?	1.21 (0.49–3.01)	0.79 (0.56–1.12)
	How satisfied are you with your access to health services?	0.76 (0.32–1.78)	1.10 (0.78–1.53)
	How satisfied are you with your transport services?	0.79 (0.32–1.94)	1.22 (0.83–1.79)

^1^ Odds Ratio and 95% Confidence interval of EXP (B); *p*-value significance ** *p* < 0.01, * *p* < 0.05.

## Data Availability

These data are not publicly available due to privacy issues. However, the data presented in this study can be provided on request from the corresponding authors.

## Supplementary Materials

The following supporting information can be downloaded at: https://www.mdpi.com/article/10.3390/ijerph19074228/s1, Table S1: Background characteristics and physical activity status of the study population.
