# Supplementary material for: Role of Environmental Quality of Life in Physical Activity Status of Individuals with and without Physical Disabilities in Saudi Arabia
Source: Int J Environ Res Public Health. 2022 Apr 1;19(7):4228. doi: 10.3390/ijerph19074228 (PMC8998774; doi:10.3390/ijerph19074228)
Supplement: Supplementary file 1 [file ijerph-19-04228-s001.zip › ijerph-1626440-supplementary.pdf]

International Journal of
Environmental Research and Public Health

Supplementary Table S1. Background characteristics and physical activity status of the study population. (Univariate analysis).

Background Variables	Total (n = 359)	MET-Minutes Less Than 600 [1]	MET-Minutes More Than 600 [2]	*p* value
	n (%)	n (%)	n (%)	
Total	359(100)	194(54)	165(46)	
**Gender**				
Males	114(31.8)	56(28.9)	58(35.2)	0.20
Females	245(68.2)	138(71.1)	107(64.8)	
**Age (years)**				
Young(15-35)	214(59.6)	121(62.4)	93(56.4)	
Middle age(36-55)	94(26.2)	46(23.7)	48(29.1)	0.46
Elder(55+)	51(14.2)	27(13.9)	24(14.5)	
**Education level**				
No education	31(8.6)	19(9.8)	12(7.3)	
Primary/Secondary	168(46.8)	89(45.9)	79(47.9)	0.69
College/University	160(44.6)	86(44.3)	74(44.8)	
**Marital status**				
Unmarried	182(50.7)	100(51.5)	82(49.7)	0.73
Married	177(49.3)	94(48.5)	83(50.3)	
**Disability status**				
Non-disabled	243(67.7)	135(69.6)	108(65.5)	0.40
Disabled	116(32.3)	59(30.4)	57(34.5)	
**Chronic disease**				
No	270(75.2)	152(78.4)	118(71.5)	0.14
Yes	89(24.8)	42(21.6)	47(28.5)	

[1] Not meeting WHO recommendation for physical activity; [2] Meeting WHO recommendation for physical activity.
